# Unsupervised machine learning models reveal two distinct post-operative physical activity profiles among joint arthroplasty patients: a United Kingdom biobank cohort study

**DOI:** 10.1186/s42836-025-00339-6

**Published:** 2025-11-12

**Authors:** Omar Musbahi, Sara Sousi, Ahmed Al-Saadawi, Sevasti Panagiota Glynou, Alex Bottle, Justin P. Cobb, Gareth G. Jones

**Affiliations:** 1 MSk Lab, Sir Michael Uren Hub, White City Campus, Imperial College London, London, W12 0BZ UK; 2 School of Public Health, White City Campus, Imperial College London, London, W12 0BZ UK; 3 School of Medicine, Faculty of Medicine and Dentistry, Queen Mary University of London, London, E1 2AD UK

## Abstract

**Objective:**

To identify and characterise distinct post-operative physical activity profiles in joint arthroplasty patients.

**Methods:**

This cohort study utilised wrist-worn accelerometer data from the UK Biobank, linked to hospital records, to identify patients who underwent primary unilateral hip or knee arthroplasty. Daily step counts from 4 to 12 months post-operatively were extracted using validated algorithms. Principal component analysis (PCA) was applied to demographic and clinical variables to reduce dimensionality, followed by clustering using k-means and Partitioning Around Medoids (PAM). The optimal number of clusters was determined using the elbow method and silhouette score. Clustering validity was assessed using the Rand Index and Adjusted Rand Index.

**Results:**

237 patients were included, the majority of whom were female, with a mean age of 66 years. Based on the elbow plot and the highest average silhouette width, a two-cluster solution was deemed optimal, consistently emerging across both clustering methods as distinct high-and low-performing physical activity profiles. High performers had significantly higher daily step counts (mean > 10,000 vs. < 6,000, *P* < 0.001), were younger, had a lower body mass index, fewer comorbidities, and were more likely to have undergone total hip replacement. Sociodemographic factors such as higher educational attainment and lower deprivation index were also associated with the high-performing group. The clustering methods demonstrated a weak-but-positive agreement (ARI = 0.224).

**Conclusion:**

Unsupervised learning of accelerometer-derived physical activity data revealed two clinically meaningful recovery profiles following joint arthroplasty. These findings underscore the multifactorial nature of post-operative recovery and support the development of personalised rehabilitation strategies to improve outcomes in lower limb arthroplasty patients.

Video Abstract

**Supplementary Information:**

The online version contains supplementary material available at 10.1186/s42836-025-00339-6.

## Introduction

Hip and knee arthroplasty are among the most commonly performed procedures worldwide [1]. Due to the ageing population, demand for hip and knee arthroplasty has surged in the United Kingdom (UK) in recent years, with further increases of up to 40% projected by 2060 [2]. Most commonly indicated for end-stage osteoarthritis (OA), this procedure is typically reserved for patients who are functionally disabled by the condition and are no longer responding to non-operative management [3]. According to White et al., only 6% of males and 5% of females met the 2008 Physical Activity Guidelines for Americans, which recommended at least 150 min of moderate-intensity physical activity per week [4]. Such limitations are associated with an increased mortality risk and exacerbation of comorbidities, primarily cardiovascular and metabolic conditions [5, 6]. In this context, joint replacement can deliver transformative outcomes for patients, with significant improvements not only in pain and function, but also in quality of life, and potentially even the halting or reversal of comorbidities [7, 8].

However, data on physical activity levels and trajectories following lower limb joint arthroplasty remain inconsistent, despite expectations of post-operative improvement. Bin Sheeha et al., in a cohort of 33 osteoarthritis patients who underwent total knee arthroplasty, found that stepping time, number of steps, and moderate-to-vigorous physical activity were all significantly increased (*P* < 0.0001) at 12 months post-operatively [9]. In contrast, a systematic review by Hammett et al. highlighted no significant improvement in physical activity at 6-months (Mean Difference: 0.14; [95% CI: −0.05–0.34]; *P* > 0.05) following hip or knee arthroplasty, and only a small, but significant, effect at 12-months post-operatively (Mean Difference: 0.43; [95% CI: 0.22–0.64]; *P* < 0.05) [10].

Given the mixed evidence, it is clear that physical activity trajectories vary substantially between individuals undergoing lower limb joint arthroplasty. However, the heterogeneity in these outcomes suggests the presence of underlying subgroups of patients with distinct recovery patterns. Whilst traditional statistical methods may fail to capture this complexity, a machine learning clustering model could be used to uncover latent patterns in physical activity behaviour and to identify clinically meaningful subgroups based on accelerometer-derived data. This study aims to look for these possible subgroups and to examine the patient-level factors influencing post-operative physical activity across different types of lower limb joint replacement using data from the UK Biobank registry.

## Methods

### Study design and data source

**Fig. 1:**
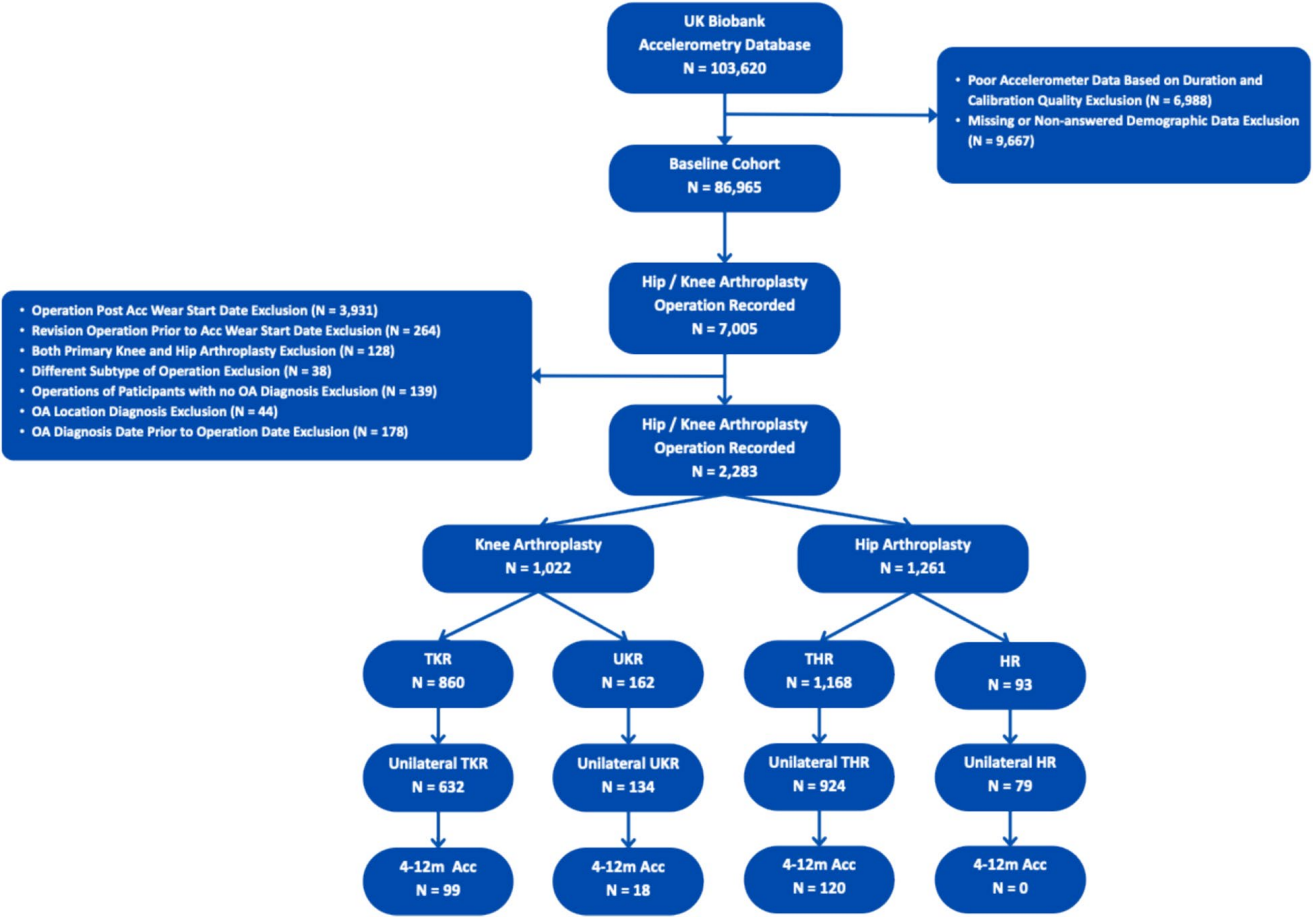
Flow diagram of participant selection

This study employed a retrospective cohort design utilising data from the UK Biobank (UKB) Accelerometery Dataset. The UKB is a large-scale database containing in-depth health information from half a million UK volunteer participants aged 40–69 years at recruitment (2006–2010). Between 2013 and 2015, participants (*n* = 103,712) wore wrist-worn triaxial accelerometers (Axivity AX3) for seven consecutive days, providing objective measurements of physical activity [11]. This accelerometery data was linked to England’s national hospital administrative database, Hospital Episode Statistics, allowing for the identification of participants who underwent primary hip or knee arthroplasty procedures and the analysis of their post-operative physical activity patterns. The UKB study received ethical approval from the North West Multi-centre Research Ethics Committee (REC reference: 11/NW/0382), and all participants provided written informed consent for data collection, analysis, and linkage to their health records (Fig. 1).

### Study population and eligibility criteria

Participants were included in the study if they had undergone primary unilateral total knee replacement (TKR), unicompartmental knee replacement (UKR), total hip replacement (THR), and hip resurfacing (HR) procedures and had valid accelerometery data available from 4 to 12 months post-operatively. This window was chosen as studies have shown that 4-month gait parameters are similar to 12-month gait parameters. Furthermore, joint replacement surgeries show recovery 3 months post-operatively [12]. The identification of arthroplasty procedures was performed using relevant OPCS-4 codes from linked hospital episode statistics.

Exclusion criteria were applied systematically according to a predefined flowchart. Participants were excluded if they had: 1) bilateral joint replacements; 2) revision procedures rather than primary arthroplasty; 3) accelerometery data collected beyond the first 12 post-operative months; 4) insufficient valid accelerometery data (defined as less than 72 h of wear time with a minimum of 16 h per day), in accordance with established quality control protocols [13]; or 5) missing data on key clinical and demographic variables.

Step count data were derived using the OxWearables step count package (v3.11.0) [14, 15], which processes raw triaxial acceleration data from wrist-worn devices. The algorithm employs a self-supervised machine learning model (ResNet18), trained and fine-tuned on data from the Axivity AX3 device used in the UKB, to detect walking periods and estimate step counts. This package has been previously used in other UKB studies [16, 17]. The model processes 5-s windows of raw acceleration data to identify walking activity and estimate step counts. The model has been validated in large-scale cohorts and has previously demonstrated robust performance in free-living conditions [11]. Adjusted step counts, which impute missing values using mean timepoint estimates across available days, were used as the final physical activity outcome in this study.

### Principal component analysis & clustering

Two types of unsupervised machine learning, partition-based clustering algorithms, were employed to identify subgroups of participants based on their demographics and physical activity (i.e., adjusted daily step count), namely, k-means and partitioning around medoids (PAM) clustering. These two algorithms were selected because they are well-established, interpretable, and computationally efficient methods for clustering mixed-type or continuous data following dimensionality reduction via Principal Component Analysis (PCA). K-means is commonly used when Euclidean distance is appropriate, whereas PAM offers improved robustness to noise and outliers by selecting representative medoids rather than centroids. PCA was undertaken to reduce the dimensionality of the selected clinical and demographic variables. To ensure the data were suitable for PCA, the following checks were performed: Bartlett’s test of sphericity and the determinant of the correlation matrix. As both categorical and continuous variables were used, categorical variables were one-hot encoded, and continuous variables were scaled to z-scores.

PCA was employed, and the loading and eigenvalues were extracted from the PCA object. The number of principal components (PCs) retained contributed to at least 80% of the variance. The variables that the PCA was applied on included age, body mass index (BMI), sex, ethnicity, smoking status, alcohol consumption, education, deprivation index, and Charlson Comorbidity Index (CCI), using a 5-year lookback window from the date the participant began wearing the accelerometer [16, 18], surgery type, time from operation to accelerometer worn, accelerometer wear season, and adjusted daily step count. The number of PCs contributing to at least 80% of the variance was used for clustering, in line with common practice [19, 20].

To determine the optimal number of clusters (*k*), both the elbow method and silhouette analysis were employed. For each clustering approach (k-means and PAM), the total within-cluster sum of squares (WSS) was plotted against a range of *k* values to produce the elbow plot, while average silhouette width was calculated to assess cluster cohesion and separation. The optimal number of clusters was selected by collectively considering the results from both the elbow plot and the silhouette plot. The “elbow” point, where the rate of decrease in WSS noticeably flattens, suggests an optimal balance between model simplicity and explained variance. In contrast, the silhouette plot displays the average silhouette width for each *k*, which reflects how well-separated and internally cohesive each cluster is; values closer to 1 indicate better-defined clustering. The higher the average silhouette score, the better the data fit for that number of clusters.

### Clustering comparison

To evaluate the similarity between the classification of the patients into the clusters by k-means and PAM, the Adjusted Random Index (ARI) was calculated. The ARI was chosen over the Rand Index (RI), as RI does not account for chance agreement and can be inflated in cases of random clustering. The ARI ranges from −1 to 1, with 1 indicating perfect agreement, 0 indicating that the clustering is no better than random assignment, and < 0 indicating worse-than-random clustering. In addition to the ARI, the Davies–Bouldin Index (DBI) was evaluated to assess the quality of clustering. The DBI measures both intra-cluster similarity (compactness) and inter-cluster difference (separation), with lower values indicating better-defined and more distinct clusters.

Post clustering, the distribution of the clinical and demographic metrics across the clusters was examined. Descriptive statistics were used to summarise the characteristics of participants within each identified cluster. To assess differences between clusters, bivariate statistical tests were performed depending on the type and distribution of the variables. The Wilcoxon rank-sum test was used for continuous variables that were not normally distributed, while Pearson’s Chi-squared test was applied to categorical variables with sufficient expected counts. For categorical variables with small cell counts, Fisher’s exact test was used to ensure accuracy in significance testing. Lastly, we compared how the two algorithms classified the patients to identify any emerging patterns based on the physical activity outcomes and patient characteristics of each cluster.

## Results

In total, 237 patients undergoing lower limb joint replacement were included in our analysis based on our inclusion and exclusion criteria: 120 underwent THR, 99 TKR, and 18 UKR. Although HR was included in the study design, no patients underwent unilateral HR and wore the accelerometer within their first post-operative year.

### Patient demographics

The cohort consisted of middle-aged patients (mean age 66.0), who were predominantly female (59%), Caucasian (98%), and, on average, overweight (mean BMI 28.9). More than half of the cohort reported alcohol consumption more than 3 days per week, and more than half had a history of smoking, with 5.5% identified as current smokers. Furthermore, the majority of patients had attained either further (45%) or higher education (40%). The baseline patient demographics can be seen in Table 1.

**Table 1:** Participant demographics of the total cohort undergoing primary lower limb arthroplasty

	***N* = 237**^*a*^
**Average Adjusted Daily Step Count**	8,053 (5,956, 10,672) Mean = 8,380
**Age**	67.0 (63.0, 70.0) Mean = 66.0
**BMI**	28.0 (25.3, 31.6) Mean = 28.9
**Sex**	
Female	141 (59%)
Male	96 (41%)
**Ethnicity**	
Non-White	4 (1.7%)
White	233 (98%)
**CCI Score**	
0	182 (77%)
1	32 (14%)
2	15 (6.3%)
3 +	8 (3.4%)
**Alcohol Consumption**	
Never	11 (4.6%)
< 3 d/w	96 (41%)
+ 3 d/w	130 (55%)
**Smoking Status**	
Never	111 (47%)
Previous	113 (48%)
Current	13 (5.5%)
**Operation Type**	
THR	120 (51%)
UKR	18 (7.6%)
TKR	99 (42%)
**Time from Operation to Accelerometer (months)**	8.37 (6.53, 9.79) Mean = 8.12
**Education**	
School leaver	35 (15%)
Further education	107 (45%)
Higher education	95 (40%)
**Deprivation Index**	
1	68 (29%)
2	48 (20%)
3	43 (18%)
4	45 (19%)
5	33 (14%)
**Wear Season**	
Autumn	73 (31%)
Winter	46 (19%)
Spring	56 (24%)
Summer	62 (26%)

*CCI *Charlson Comorbidity Index, *THR *Total Hip Replacement, *UKR *Unicompartmental Knee Replacement, *TKR *Total Knee Replacement

^*a*^Median (IQR); *n* (%)

### Principal component analysis

**Fig. 2:**
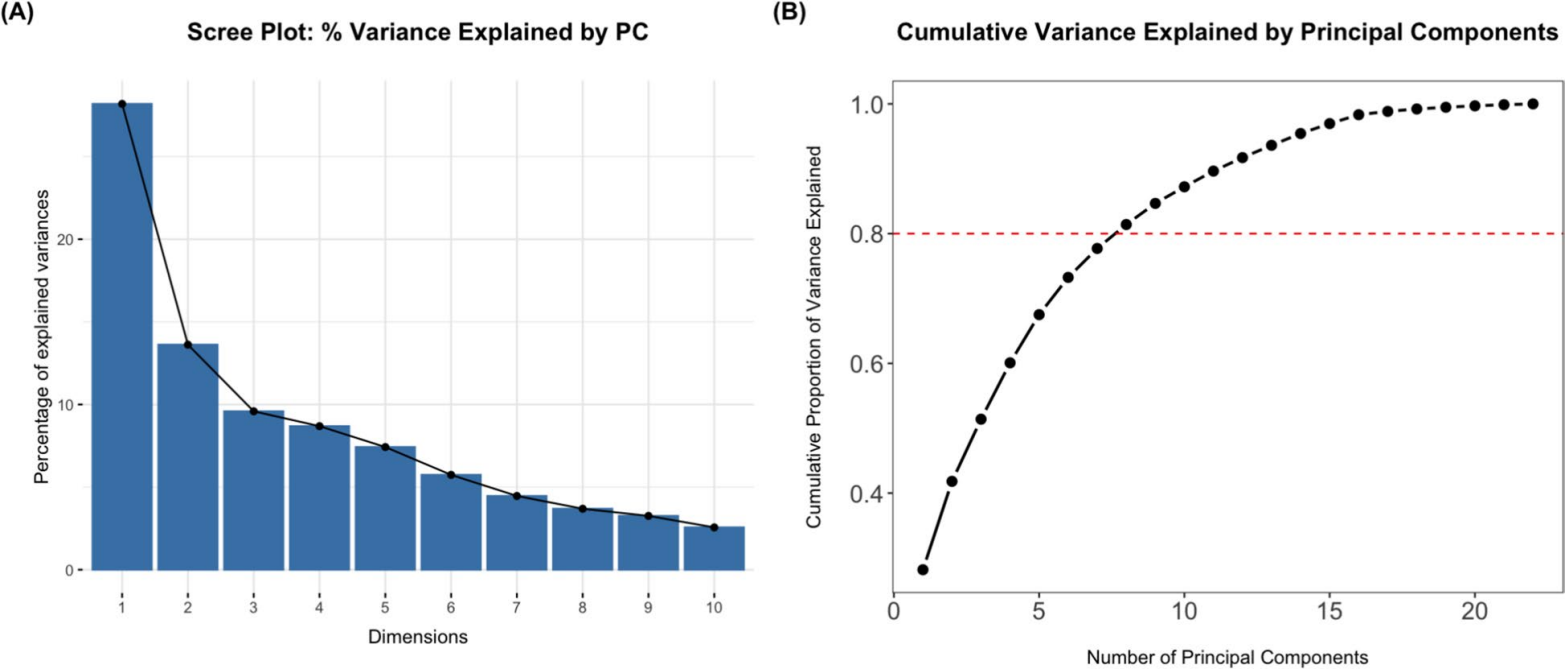
Principal component analysis (PCA) variance explanation plots. **A** Scree plot; **B** Line plot. (The red dashed line indicates the 80% cumulative variance cutoff. PC: Principal Component)

The scree plot shows the proportion of variance explained by each PC (Fig. 2A). It demonstrates that the percentage of explained variance declines sharply up to the third PC, after which the rate slows down considerably. The line plot displays the cumulative proportion of variance explained across successive PCs. Of the 22 PCs, 8 were retained as they cumulatively accounted for 81.4% of the total variance (Fig. 2B). To improve the interpretability of the clustering results, a PCA loadings plot was generated (Supplementary Fig. S1). This illustrates the contribution of each original variable to the top eight PCs, which together explained over 80% of the variance in the dataset. Variables such as adjusted daily step count, age, CCI score, BMI, and time from surgery to accelerometer wear were among the most influential across the retained components.

### Clustering analysis

**Fig. 3:**
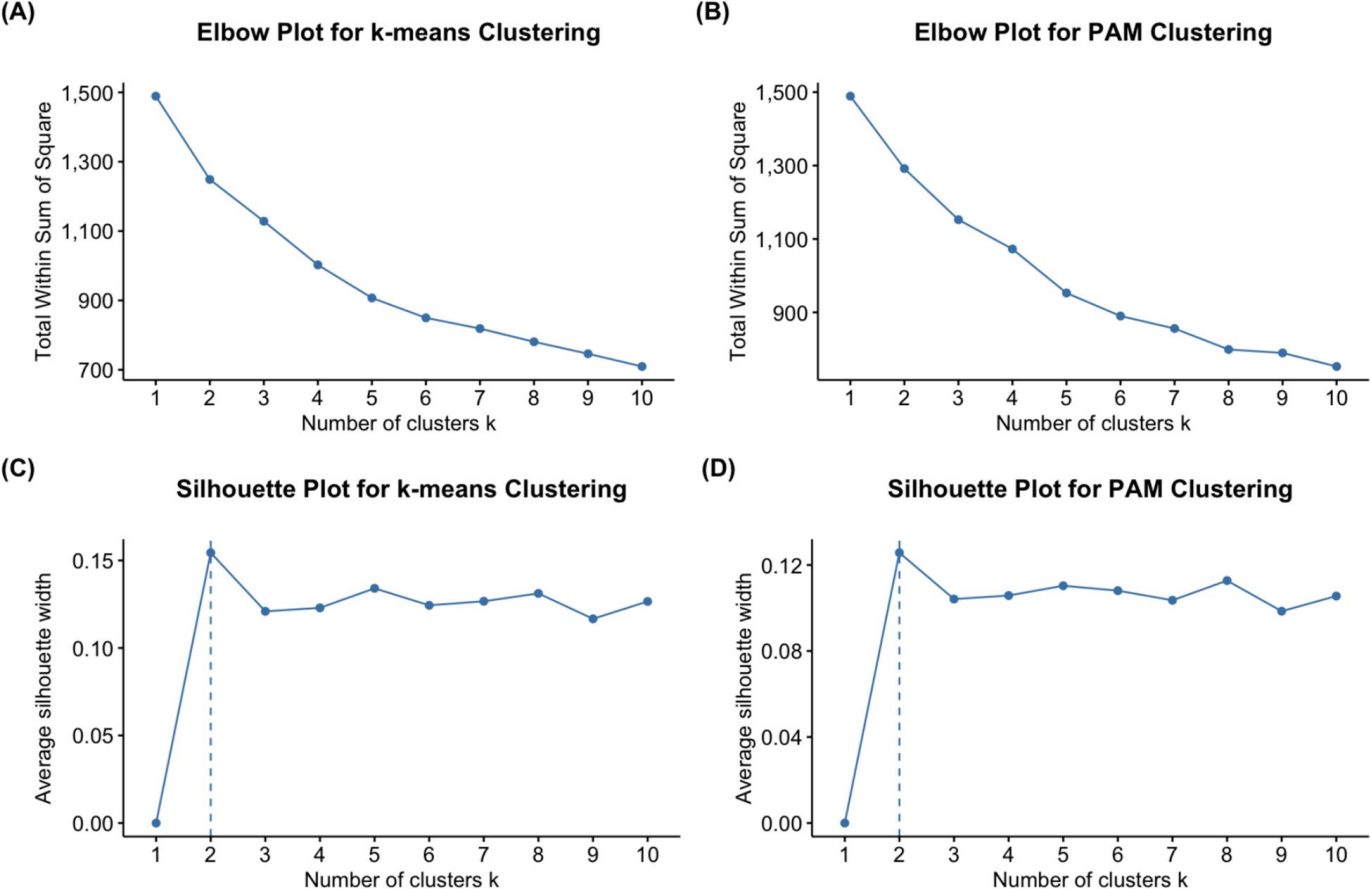
Comparison of clustering performance using k-means and PAM algorithms. **A** Elbow plot for k-means; **B** Elbow plot for PAM; **C** Silhouette plot for k-means; **D** Silhouette plot for PAM. (PAM: Partitioning Around Medoids)

**Fig. 4:**
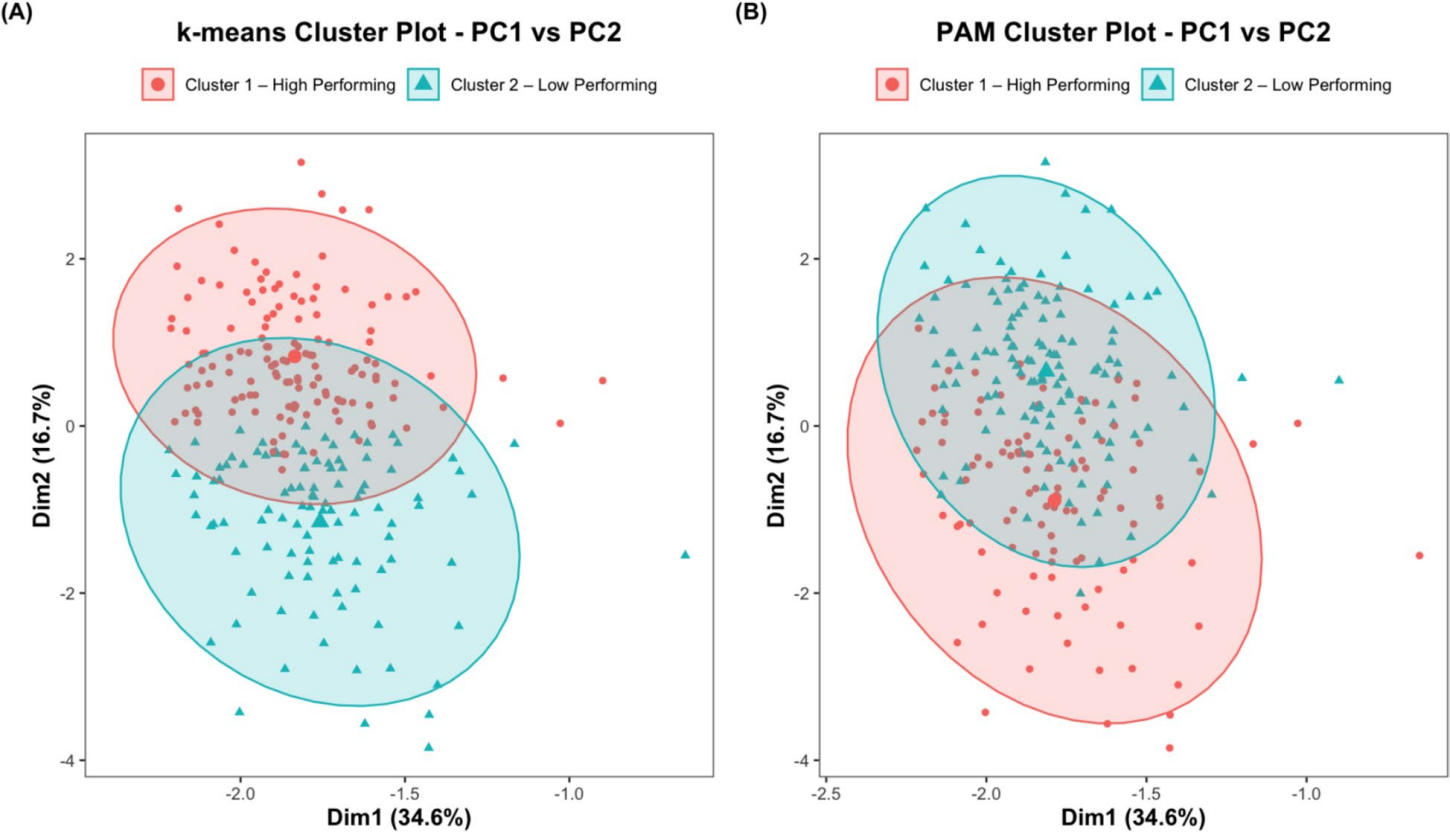
Cluster visualisation of average daily step count profiles using (**A**) k-means and (**B**) PAM clustering. (The ellipses indicate 95% confidence regions based on a normal distribution. PAM: Partitioning Around Medoids, PC: Principal Component Cluster 1: “High Performing”, Cluster 2: “Low Performing”)

The elbow plot displays the total WSS across different cluster counts. For both k-means and PAM clustering, it did not reveal a noticeable decrease in WSS (Fig. 3A and B). Furthermore, the silhouette plot indicated the highest average silhouette width, suggesting the optimal number of clusters (*k*) to be used. In Fig. 3C and D, the highest average silhouette width for both clustering techniques was observed at *k* = 2. As a result, the patient cohort was separated into two clusters: high-performing and low-performing, based on their average post-operative daily step count profiles (Fig. 4).

**Fig. 5:**
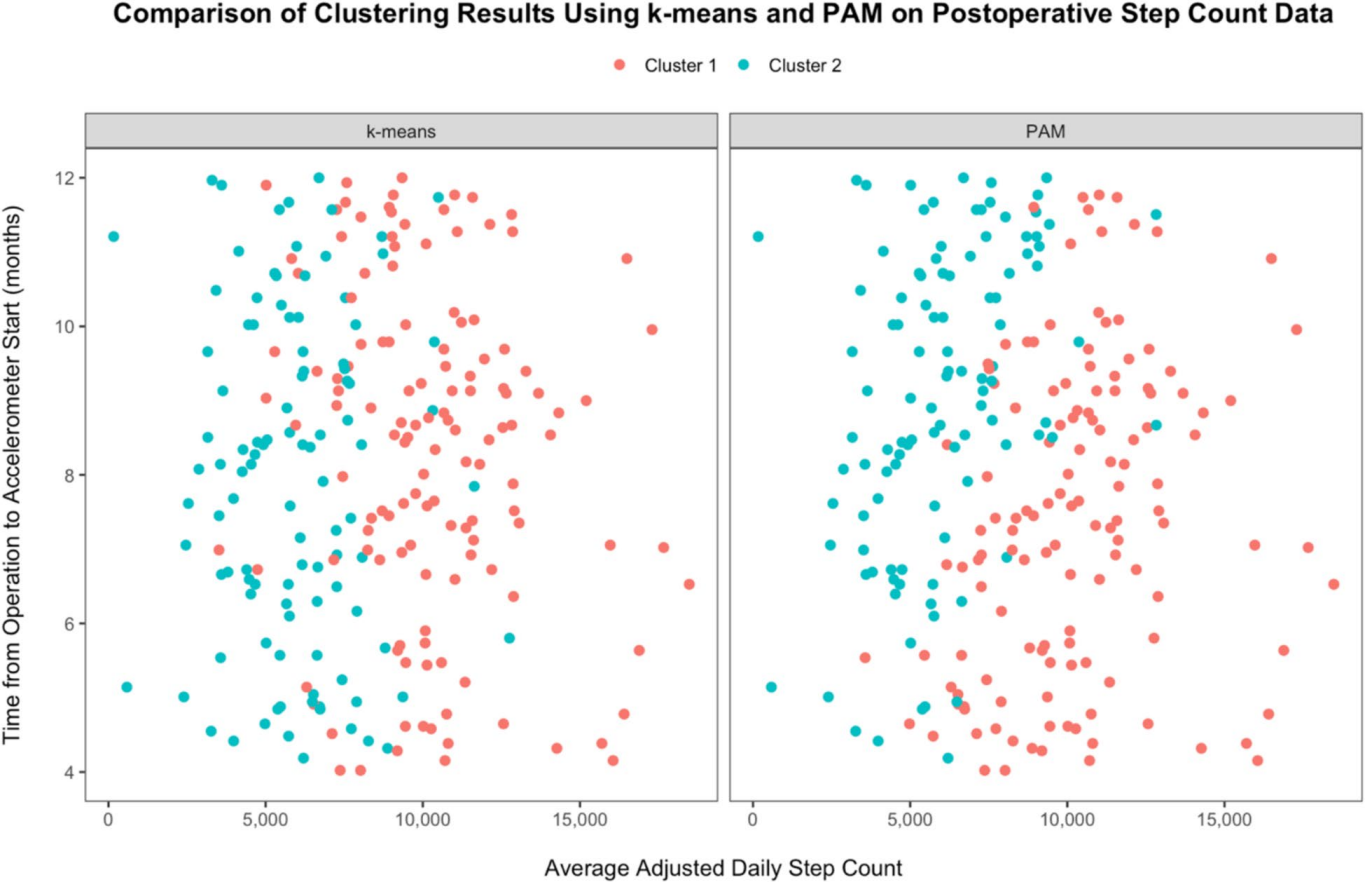
Cluster assignments displaying average adjusted daily step count against time from operation to accelerometer start. (Cluster 1: Red, Cluster 2: Blue)

Table 2 reports the patient characteristics within each cluster, stratified by the two distinct clustering techniques employed. There is a clear average daily step count difference between the high-performing cluster and the low-performing cluster across both k-means and PAM clustering methods (*P* < 0.001) (Fig. 5). Additionally, age and BMI were all significantly lower in the high-performing clusters compared with their counterparts (*P* < 0.001) for both clustering techniques, while alcohol consumption was significantly higher only for k-means (*P* < 0.001). Using the CCI, the proportion of patients with comorbidities was also significantly lower in the high-performing cluster (*P* < 0.001). One notable difference in patient demographics between the clusters generated by k-means and PAM methods was education, which was significantly different between the high- and low-performing clusters in k-means (*P* < 0.001), but not in PAM clustering (*P* = 0.076). Similar patterns were observed with operation type in k-means (*P* < 0.001) and PAM clustering (*P* = 0.1). Furthermore, in k-means, there was a higher proportion of UKRs and a lower proportion of TKRs in the high-performing group, compared with the low-performing cluster. Overall, there was a weak-but-positive agreement between the clustering performance of both methods, as demonstrated by an ARI of 0.224. The BDI was 0.867 for k-means, indicating relatively well-defined clusters, and 1.134 for PAM, suggesting slightly less compact and more overlapping clusters.

**Table 2:** Patient demographics in each cohort using two separate clustering techniques

	**Cluster 1-High Performing**	**Cluster 2-Low Performing**	***P*-value**^***b,3***^	**Cluster 1-High Performing**	**Cluster 2-Low Performing**	***P*-value**^***b,3***^
	***N* = 133**^***a***^	***N* = 046**^***a***^		***N* = 131**^***a***^	***N* = 106**^***a***^	
**Average Adjusted Daily Step Count**	10,101 (8,705, 11,615) Mean = 10,307	5,773 (4,503, 7,265) Mean = 5,916	< 0.001***	10,136 (8,271, 11,631) Mean = 10,313	5,773 (4,528, 7,539) Mean = 5,992	< 0.001***
**Age**	66.0 (61.0, 69.0) Mean = 65.0	68.0 (65.0, 71.0) Mean = 67.3	0.001**	65.0 (60.0, 69.0) Mean = 64.3	69.0 (65.0, 72.0) Mean = 68.2	< 0.001***
**BMI**	26.1 (24.0, 28.0) Mean = 26.4	31.5 (29.0, 35.0 Mean = 32.2	< 0.001***	27.2 (24.3, 31.0) Mean = 28.2	29.0 (26.6, 33.1) Mean = 29.8	0.004**
**Sex**			0.117			0.107
Female	85 (64%)	56 (54%)		84 (64%)	57 (54%)	
Male	48 (36%)	48 (46%)		47 (36%)	49 (46%)	
**Ethnicity**			> 0.999			> 0.999
Non-White	2 (1.5%)	2 (1.9%)		2 (1.5%)	2 (1.9%)	
White	131 (98%)	102 (98%)		129 (98%)	104 (98%)	
**CCI Score**			< 0.001***			< 0.001***
0	119 (89%)	63 (61%)		122 (93%)	60 (57%)	
1	10 (7.5%)	22 (21%)		7 (5.3%)	25 (24%)	
2	3 (2.3%)	12 (12%)		1 (0.8%)	14 (13%)	
3 +	1 (0.8%)	7 (6.7%)		1 (0.8%)	7 (6.6%)	
**Alcohol Consumption**			< 0.001***			0.575
Never	5 (3.8%)	6 (5.8%)		6 (4.6%)	5 (4.7%)	
< 3 d/w	41 (31%)	55 (53%)		49 (37%)	47 (44%)	
+ 3 d/w	87 (65%)	43 (41%)		76 (58%)	54 (51%)	
**Smoking Status**			0.463			0.983
Never	67 (50%)	44 (42%)		62 (47%)	49 (46%)	
Previous	59 (44%)	54 (52%)		62 (47%)	51 (48%)	
Current	7 (5.3%)	6 (5.8%)		7 (5.3%)	6 (5.7%)	
**Operation Type**			< 0.001***			0.1
THR	82 (62%)	38 (37%)		67 (51%)	53 (50%)	
UKR	11 (8.3%)	7 (6.7%)		14 (11%)	4 (3.8%)	
TKR	40 (30%)	59 (57%)		50 (38%)	49 (46%)	
**Time from Operation to Accelerometer (months)**	8.60 (6.86, 9.79) Mean = 8.28	8.06 (6.13, 9.73) Mean = 7.91	0.189	7.35 (5.54, 9.10) Mean = 7.40	9.13 (7.62, 10.81) Mean = 9.01	< 0.001***
**Education**			< 0.001***			0.076
School leaver	15 (11%)	20 (19%)		14 (11%)	21 (20%)	
Further education	49 (37%)	58 (56%)		58 (44%)	49 (46%)	
Higher education	69 (52%)	26 (25%)		59 (45%)	36 (34%)	
**Deprivation Index**			0.121			0.879
1	46 (35%)	22 (21%)		39 (30%)	29 (27%)	
2	24 (18%)	24 (23%)		29 (22%)	19 (18%)	
3	19 (14%)	24 (23%)		23 (18%)	20 (19%)	
4	24 (18%)	21 (20%)		23 (18%)	22 (21%)	
5	20 (15%)	13 (13%)		17 (13%)	16 (15%)	
**Wear Season**			0.086			0.357
Fall	40 (30%)	33 (32%)		37 (28%)	36 (34%)	
Winter	23 (17%)	23 (22%)		22 (17%)	24 (23%)	
Spring	27 (20%)	29 (28%)		34 (26%)	22 (21%)	
Summer	43 (32%)	19 (18%)		38 (29%)	24 (23%)	

*Abbreviations: CCI *Charlson Comorbidity Index, *THR *Total Hip Replacement, *UKR *Unicompartmental Knee Replacement, *TKR *Total Knee Replacement

^3^*P < 0.05; **P < 0.01; ***P < 0.001

^a^Median (IQR); *n* (%)

^b^Wilcoxon rank sum test; Pearson’s Chi-squared test; Fisher’s exact test

## Discussion

This study utilises machine learning to directly explore the different post-operative recovery profiles and patient factors influencing physical activity levels following lower limb joint arthroplasty. Our study highlights several crucial findings, most notably the identification of two distinct clusters through the elbow and silhouette plot. These clusters represented two primary post-arthroplasty physical activity profiles, categorised into a high-performing and low-performing cluster. The clustering agreement between the two approaches, k-means and PAM, was weak but positive, with an ARI of 0.224. Patients in the high-performing cluster, with higher average step counts, were significantly younger, had lower BMI, and fewer comorbidities compared with those in the low-performing cluster. Furthermore, various behavioural and socioeconomic factors were found to influence post-operative physical activity levels. These included significantly higher alcohol consumption, greater educational attainment, and lower deprivation index in the high-performing cluster.

Several aspects of our study distinguish it from prior research. First, our study offers a comprehensive overview of functional outcomes after lower limb joint arthroplasty and the contributing factors that may influence them, which is particularly important in the context of OA. Second, the incorporation of clustering analysis provides unique insights into recovery patterns following arthroplasty. It highlights the multifactorial nature of physical functioning post-operatively, which is influenced not only by physical factors such as age and BMI, but also by the patient’s broader social context. To ensure robustness in our analysis, we implemented two distinct unsupervised machine learning algorithms for comparison, namely k-means and PAM clustering. The ARI indicated a weak-but-positive agreement between the findings of both techniques, enhancing the validity of our findings.

Our findings regarding the patient factors influencing post-operative physical activity levels are in agreement with the existing literature. For instance, Paxton et al. similarly reported that patients with a higher BMI (THR: −7.1 min/week, *P* = 0.003; TKR: −5.9 min/week, *P* = 0.02) and comorbidities, such as renal failure (TKR: −17 min/week, *P* = 0.003) and neurological disorders (TKR: −30 min/week, *P* = 0.006), were associated with a smaller change in physical activity levels from pre- to post-operatively following TKR and THR [21]. However, female gender was found to be significantly associated with lower physical activity levels compared with males following TKR (−9.1 min/week, *P* = 0.04), which contrasts with our findings [21]. An important methodological difference exists between the two studies, whereby self-reported active minutes were utilised as their metric of PA, unlike our use of objectively measured data from the wrist-worn accelerometer [21]. Several studies have suggested that self-reported physical activity often provides unreliable estimates of actual performance compared with objective methods [22, 23]. This potentially limits the validity of the aforementioned study’s findings. Another retrospective study by Williams et al. reported that lower age, BMI, and male gender were all independently associated with increased participation in sports following hip and knee arthroplasty (*P* < 0.001) [24]. While their study does not focus on routine low-level physical activity, it still provides a useful indication of the various factors influencing PA post-operatively. Additionally, similar to the previous study, they relied solely on a patient-reported questionnaire as their measure of physical activity levels, which highlights a key strength of our study in comparison [24]. One potential predictor considered in the two aforementioned studies that our study failed to evaluate is pre-operative physical activity levels [21, 24]. A comparison between pre- and post-operative PA levels would have provided a more accurate and complete perspective on whether post-operative PA levels were directly influenced by the arthroplasty procedure itself.

Hospitalisation and post-operative costs account for the highest proportion of the total 1-year cost for both THR and TKR [25]. When combined with the opportunity costs of functional disability secondary to OA, the rising incidence of the condition and subsequent demand for arthroplasty will only serve to exacerbate the financial strain on the National Health Service. We hope that our findings will aid clinicians in identifying patients at risk of experiencing poor rehabilitative outcomes following arthroplasty, and in turn, make the necessary adaptations to optimise their recovery. This approach could be particularly beneficial for optimising resource allocation, enabling low-cost remote or self-directed rehabilitation for low-risk, high-performing patients [26], while offering more intensive programmes for those at risk of poor recovery. More than anything, our study lays the foundations for future research to build upon these findings, intending to develop a risk stratification model that facilitates a more streamlined and efficient post-operative care pathway.

## Limitations

While our study offers several novel findings, the nature of the dataset limits their interpretation and validity. Specifically, the Biobank dataset lacked data on initial mobilisation or adherence to physiotherapy, both of which have been linked to improved patient outcomes. Additionally, the study did not assess intra-individual activity trends or causality. The limited sample size (*n* = 237), particularly when divided into subgroups, reduced statistical power, limited generalisability, and potentially may have introduced some bias. Furthermore, the modest agreement between clustering methods (ARI = 0.224) suggests potential instability in clustering definitions, which may limit the reproducibility of our study. Regarding patient demographics, the cohort was predominantly white (98.6%) and well educated, limiting the generalisability of the findings to other populations. Moreover, the average time from operation to accelerometer wear was six months, and the device was only worn for one week, representing another limitation, as it may not fully reflect long-term physical activity patterns. Lastly, another limitation is the variability in timing of accelerometery wear across the cohort post-operatively, over seven days between 4–12 months post-operatively. The heterogeneity may introduce variability in activity levels unrelated to physical activity profiles, potentially skewing the clustering results.

## Conclusion

This study is the first to apply unsupervised machine learning to objectively assess post-operative physical activity profiles in a large joint arthroplasty cohort. The elbow and silhouette plots indicated two optimal clusters, representing distinct recovery profiles: high-performing and low-performing. Between the two clustering approaches, k-means and PAM, agreement was weak but positive (ARI = 0.224). Significant demographic differences were observed between clusters, with the high-performing group being younger, having lower BMI, and fewer comorbidities. Behavioural and socioeconomic differences were also evident, including higher educational attainment and a lower deprivation index in the high-performing group. These findings highlight the heterogeneity in functional recovery following lower limb arthroplasty and underscore the importance of a personalised, data-driven approach to post-operative care. By identifying patients at risk of poor recovery trajectories, clinicians may be better positioned to tailor interventions and optimise long-term outcomes. Future research should explore the integration of pre-operative activity levels and rehabilitation adherence to further refine these predictive models.

## Supplementary Information

Supplementary Material 1: Fig. S1. PCA Loadings for Top Principal Components.

## Data Availability

No datasets were generated or analysed during the current study.
