# Supplementary material for: Unsupervised machine learning models reveal two distinct post-operative physical activity profiles among joint arthroplasty patients: a United Kingdom biobank cohort study
Source: Arthroplasty. 2025 Nov 12;7:57. doi: 10.1186/s42836-025-00339-6 (PMC12606873; doi:10.1186/s42836-025-00339-6)
Supplement: Supplementary file 2 — Supplementary Material 1: Fig. S1. PCA Loadings for Top Principal Components. [file 42836_2025_339_MOESM1_ESM.docx]

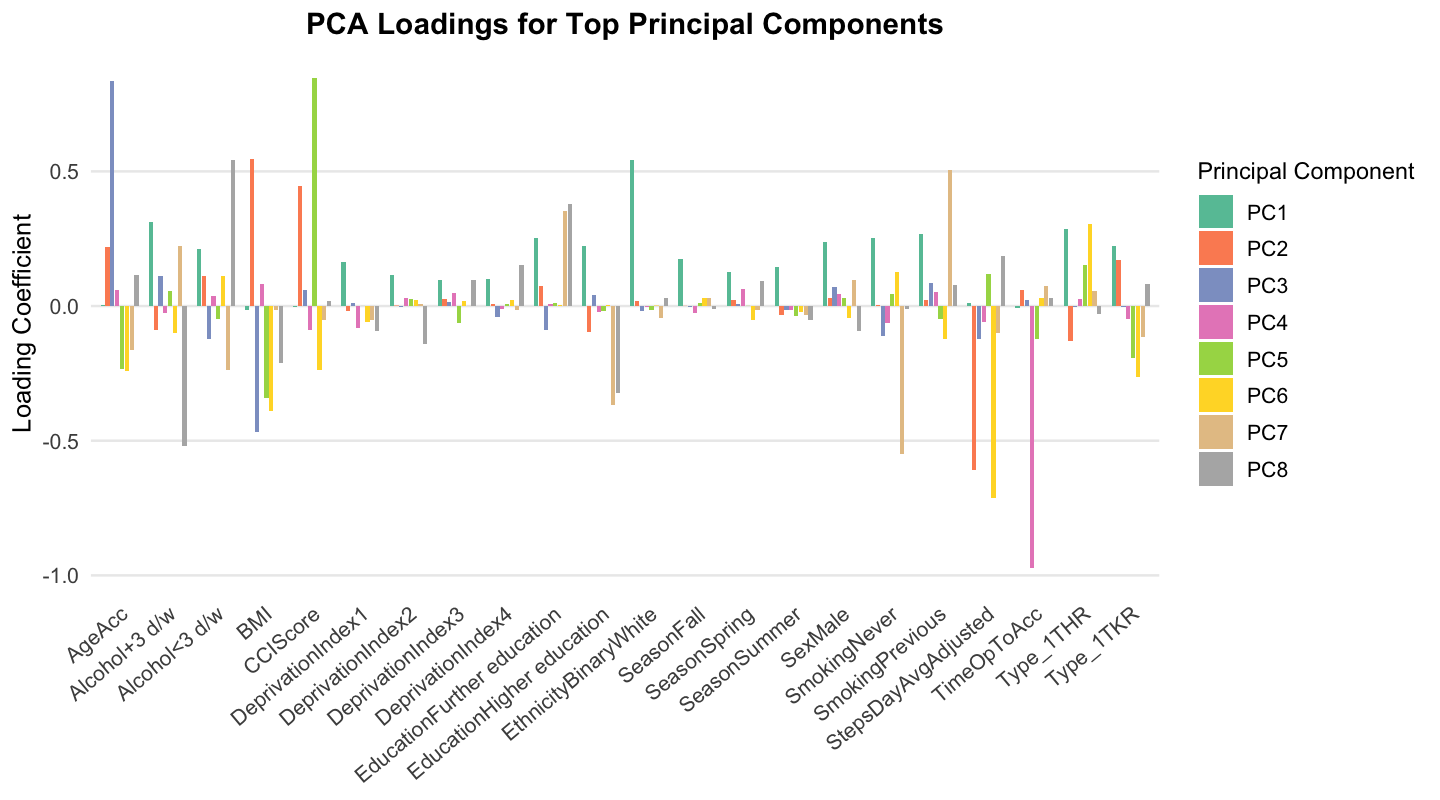


**Supplementary Figure 1:** **PCA Loadings for Top Principal Components**

Bar plot illustrating the loading coefficients of each original variable across the top eight principal components (PC1–PC8), which collectively explain ≥80% of the total variance in the dataset. Higher absolute loading values indicate stronger contributions of the variable to the corresponding component.
